# Registration Techniques for Clinical Applications of Three-Dimensional Augmented Reality Devices

**DOI:** 10.1109/JTEHM.2020.3045642

**Published:** 2020-12-17

**Authors:** Christopher M. Andrews, Alexander B. Henry, Ignacio M. Soriano, Michael K. Southworth, Jonathan R. Silva

**Affiliations:** 1 Department of Biomedical EngineeringWashington University in St Louis, McKelvey School of Engineering 7284 St LouisMO63130USA; 2 SentiAR, Inc.St. LouisMO63108USA

**Keywords:** Augmented reality (AR), HoloLens, medical imaging, image registration, surgery

## Abstract

Many clinical procedures would benefit from direct and intuitive real-time visualization of anatomy, surgical plans, or other information crucial to the procedure. Three-dimensional augmented reality (3D-AR) is an emerging technology that has the potential to assist physicians with spatial reasoning during clinical interventions. The most intriguing applications of 3D-AR involve visualizations of anatomy or surgical plans that appear directly on the patient. However, commercially available 3D-AR devices have spatial localization errors that are too large for many clinical procedures. For this reason, a variety of approaches for improving 3D-AR registration accuracy have been explored. The focus of this review is on the methods, accuracy, and clinical applications of registering 3D-AR devices with the clinical environment. The works cited represent a variety of approaches for registering holograms to patients, including manual registration, computer vision-based registration, and registrations that incorporate external tracking systems. Evaluations of user accuracy when performing clinically relevant tasks suggest that accuracies of approximately 2 mm are feasible. 3D-AR device limitations due to the vergence-accommodation conflict or other factors attributable to the headset hardware add on the order of 1.5 mm of error compared to conventional guidance. Continued improvements to 3D-AR hardware will decrease these sources of error.

## I. Background

Spatial reasoning is one of the primary challenges of clinical interventions across medical disciplines. Many tools and workflows exist to help clinicians visualize and understand the relationship between critical anatomy and surgical tools. Image-guided procedures incorporate pre-operative imaging or real-time imaging to help physicians guide surgical tools to treatment targets and avoid damaging adjacent structures and tissue. These tools are utilized for intracardiac procedures, neurosurgery, biopsies, and many other clinical procedures. A key challenge of image-guided procedures is that information is typically displayed on a two-dimensional (2D) screen, requiring clinicians to mentally relate the images to the three-dimensional (3D) patient and to frequently switch their attention between the patient and display [1]. For a technology like fluoroscopy, the challenges of relating images to the patient orientation can result in excess ionizing radiation exposure to the patient and clinical team [2]. Spatial reasoning can be challenging even when the surgeon has direct view of the interventional target, such as tumor resection. In many cases, the boundary between the tumor and healthy tissue is not visible or palpable. In breast-conserving surgery, for example, 20-40% of patients require a second procedure because of inadequate tumor margins, which exposes patients to additional procedural risks and increases healthcare costs [3]. Many clinical workflows such as these would benefit from more direct and intuitive visualizations of anatomy, surgical plans, or other information crucial to the procedure.

**FIGURE 1.**
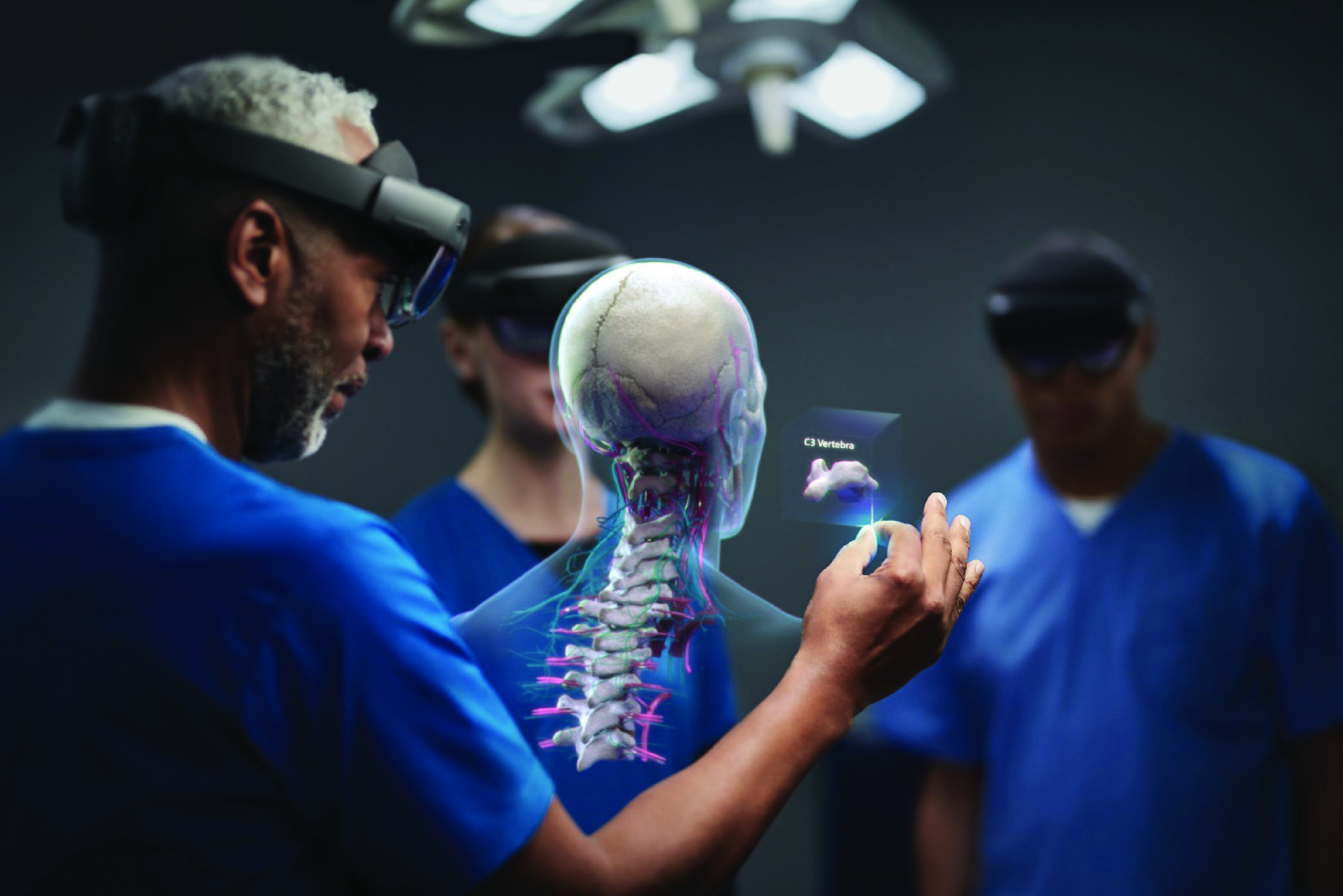
The Microsoft HoloLens 2 head-mounted three-dimensional augmented reality (3D-AR) interface. Transparent stereoscopic displays allow physicians to view 3D holographic anatomy and the physical environment simultaneously. Used with permission from Microsoft. Original source: https://news.microsoft.com/hololens2_healthcare2/.

Augmented reality (AR) is an emerging technology that has the potential to help physicians with spatial reasoning during clinical interventions [4]–[8]. The term AR broadly describes computing devices that overlay digital information onto a view of the physical world. While there are many forms of AR, including smartphone- or tablet-based displays, head-mounted 3D AR (3D-AR) devices introduce exciting new possibilities for clinical interventions. 3D-AR devices are head-worn computing devices that display virtual objects as though they are present in the user’s physical environment (**Fig. 1**). 3D-AR devices generally have two transparent displays that sit in front of the user’s eyes. Using stereoscopic rendering with the two screens, the devices display digital content that appears as 3D holograms to the user. 3D-AR devices typically incorporate on-board spatial tracking hardware and algorithms that track the headset’s movement in the physical environment. The spatial tracking allows 3D-AR devices to display holograms that appear in stable locations in users’ environments, which makes the 3D experience more immersive.

While the landscape of 3D-AR hardware is increasingly diverse, the Microsoft HoloLens (Microsoft, Redmond, WA) was one of the first commercially available 3D-AR devices. Notable hardware features of the HoloLens and other 3D-AR devices are presented in **Table 1**. The spatial tracking functionality used by the HoloLens is referred to as simultaneous localization and mapping (SLAM). The SLAM functionality can be integrated into HoloLens software, allowing applications to understand and interact with the user’s physical environment [9], [10]. As its name suggests, SLAM uses sensor data to construct a map of the environment while tracking the device’s position within the environment. This technique was first developed in the early 90’s [11] and quickly gained traction as a key component to many applications in robotics [12]. Other notable features of the HoloLens that are clinically relevant include the use of voice commands, hand gestures, and visual gaze to control HoloLens software. These features enable users to operate the HoloLens in sterile environments and are compatible with clinical situations that require the physician to use both hands for an intervention.

**TABLE 1:** Comparison of 3D-AR Device Hardware. The General-Purpose Magic Leap and HoloLens Headsets are Widely Available. The Specialized Augmedics xVision Headset has Received FDA 510(k) Clearance

	Magic Leap 1	HoloLens 1	HoloLens 2	xVision
Processor	6-core 1.7 GHz	4-core 1 GHz	8-core 2.6 GHz	Unknown
Memory	8 GB	2 GB	4 GB	Unknown
Color Camera Resolution	1.5MP	2.4MP	8MP	None
Monochrome Cameras	4	4	4	None
IR Sensor	Yes	Yes	Yes	Yes
Depth Sensor	ToF	ToF	ToF	Unknown
Weight (grams)	316	579	566	Unknown
Field of View (degrees)	50	34	52	Unknown
Resolution (per eye)	$1280 \times 960$	$1268 \times 720$	$2048 \times 1080$	Unknown
Eye Tracking	Yes	No	Yes	No
Tracking	6DoF	6DoF	6DoF	6DoF

Studies of 3D-AR devices have demonstrated their utility as clinical visualization tools. One advantage of 3D-AR displays is that they provide the user with a high degree of flexibility for positioning and scaling views of clinical data. A prospective study evaluated the use of the HoloLens as an alternative to conventional monitors for endoscopic ureteroscopy in immersive simulated procedures. The evaluation of 72 participants found that procedural times and Objective Structured Assessment of Technical Skill (OSATS, a previously validated global rating scale for ureteroscopy) scores improved with the HoloLens compared to conventional monitors. 95% of participants agreed that the HoloLens is feasible to introduce clinically and will have a role within surgery [13]. Another key advantage of 3D-AR devices is that 3D visualizations are often more intuitive than 2D visualizations. This may help clinicians more easily determine the spatial relationships between clinical tools and anatomical structures. We developed a 3D-AR visualization tool for cardiac electrophysiology (EP) procedures that provides the user with a 3D holographic view of real-time catheter positions and 3D electroanatomic maps [14]–[16]. Catheter navigation errors were significantly lower compared to conventional 2D display navigation (2.99 ± 1.91 mm vs 4.50 ± 3.74 mm, p < 0.005) [15]. Commercial applications of 3D-AR to clinical problems are also emerging. The Novarad OpenSight system for pre-surgical planning was the first HoloLens-based system to be cleared by the FDA [17].

While these results are promising for the role of 3D-AR devices as clinical visualization tools, there are many intriguing applications of 3D-AR that require displaying or overlaying important information at spatially precise locations. In a clinical context, this can mean visualizations of anatomy or surgical plans that appear directly on the patient. The accuracy of these visualizations is a key factor in determining whether they can be used to guide clinical interventions. Commercially available 3D-AR devices were designed to ensure that holograms appear spatially stable as users move around in room-sized environments. Localization errors on the order of a few centimeters are common when using these devices. While these spatial errors are acceptable for many basic AR experiences, errors of this size are incompatible with many clinical procedures. For this reason, a variety of approaches for improving 3D-AR registration accuracy have been explored. A commercial example includes the Augmedics xVision system, which uses a custom 3D-AR headset [18].

The focus of this review is on the methods, accuracy, and clinical applications of registering 3D-AR devices with the clinical environment. The works cited represent a variety of approaches for registering holograms to patients including manual registrations, computer vision-based registrations, and registrations that incorporate external tracking systems. Many of the approaches cited in this review have been developed in other fields such as robotics [19]–[21], however, their application to clinical problems with recently developed 3D-AR hardware is novel.

At the time of writing, several recent advances in AR-related hardware have yet to make an appearance in clinical settings but are sure to move clinical applications of 3D-AR forward. 3D-AR visual fidelity will benefit from improvements in display hardware (e.g. pinhole waveguides, interference-based holography, etc.), and tracking accuracy will be improved by advances in sensor hardware (e.g. Apple’s LiDAR, Microsoft’s Azure Kinect, etc.). The HoloLens 2 was recently released and will likely make an impact on clinical AR, as evidenced by a concept collaboration between Microsoft and Philips [22]. Although various head-worn stereoscopic displays are available in the consumer electronics market, there are limited peer-reviewed articles that describe the performance of 3D-AR on registered physical environments in a clinical setting. The majority of the studies to date utilized the Microsoft HoloLens, but the techniques discussed are applicable to any 3D-AR device with suitable hardware.

## II. Manual Registrations

**FIGURE 2.**
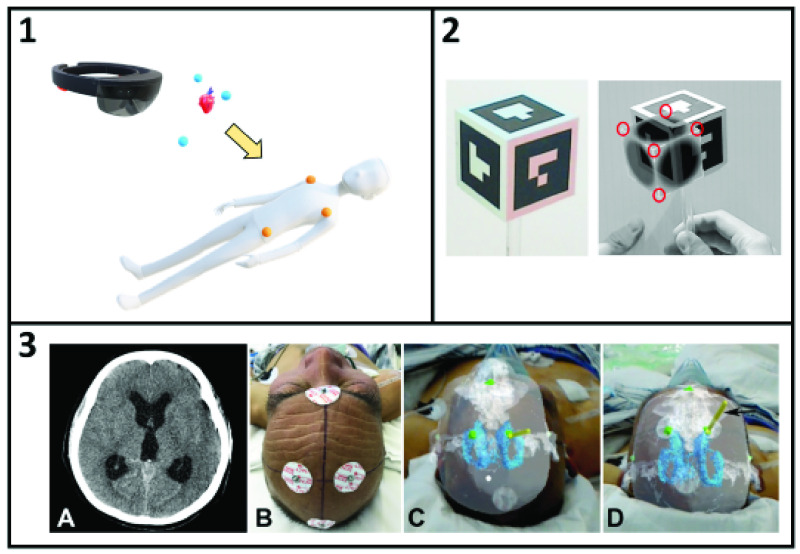
Illustration of manual hologram registration (Panel 1). Pre-operative imaging is performed and analyzed to segment relevant anatomy (the heart). Anatomical landmarks or fiducials (yellow spheres on the patient) are segmented from the imaging data (blue spheres). The user interacts with the headset software to manually translate and rotate the holographic anatomy and landmarks until the physical and holographic landmarks are aligned. Alternative interfaces have explored moving physical objects such as handheld cubes to holographic targets (Panel 2, adapted from Azimi *et al.* 2019 [28]). Manual registration was applied clinically to guide external ventricular drain insertion (Panel 3, adapted from Li *et al.* 2018 [29]). CT anatomy (A) was segmented and electrocorticography electrodes attached to the patient’s head were used as registration markers (B). The unregistered holographic anatomy (C) was manually adjusted to perform the registration (D).

The simplest approach to registering 3D-AR holograms to a patient is to manually perform the registration. The general principle is that the user sees a hologram in the headset and manually adjusts the hologram position using software controls until the hologram is spatially aligned with the physical structure it represents (**Fig. 2**, **Panel 1**). This approach was evaluated in a surgical environment by positioning a holographic view of the patient’s scapula over the patient’s shoulder during a successful reverse shoulder arthroplasty procedure [23]. In another preliminary study, holographic visualizations of computed tomography (CT) angiography scans were used to guide vascular pedunculated flap procedures [24]. Our group applied this technique to a registration of lateral skull base anatomy to 3D-printed and cadaveric skulls [25]. CT scans were processed to generate 3D anatomical geometry. Users registered the 3D anatomy using voice and hand gestures to manipulate the holograms. A similar approach was used by another group to manually align a hologram with a custom near-infrared (IR) camera calibration board so the fluorescence detected by the camera could be displayed in the headset [26]. A downside to manual registrations is that they can be tedious and difficult to perform accurately. One group developed a novel software interface to simplify the registration process for the user [27]. The method uses three fiducial points and allows the user to perform the registration in three steps, where two of the steps only require simple rotations. This method reduces some of the difficulty experienced by the user.

While manual registrations are often performed by positioning holograms over physical targets, the same result can be achieved by moving physical objects to holographic targets. A major challenge of this approach is that judging the relative depths of physical and virtual objects is very difficult. When touching physical objects, the user feels tactile feedback when an object contacts its target. Additionally, a physical object will occlude the view of another physical object behind it. This visual occlusion provides an important depth cue. Holograms have no tactile feedback, and current-generation hardware lacks the spatial mapping resolution to integrate occlusion of small objects. An interesting solution to this problem was to align holographic cubes with handheld cubes that were tracked by an external camera [28]. Study participants matched the positions of holographic cubes with handheld cubes by using the 3D shape of the cube and the edge lengths to achieve better depth alignment (**Fig. 2**, **Panel 2**). While manual registration is a simple and relatively imprecise technique, this approach has already been applied clinically in a study of bedside external ventricular drain insertion [29]. Compared to retrospectively included controls, the mean deviation from the surgical target was lower (4.34 mm vs 11.26 mm) and the number of passes required (a predictor of complications) was reduced (1.07 vs 2.33) (**Fig. 2**, **Panel 3**).

## III. Computer Vision Registration Targets

While manual registrations have proven useful, the registration process adds time to procedures and introduces the possibility of human error impacting registration accuracy. One of the primary tools available to help automate registrations is the front-facing camera on the HoloLens. This camera can record photos and videos, but it can also be utilized by computer-vision algorithms. The general principle of computer vision tracking approaches is that a 2D image or 3D object with known dimensions is tracked by a digital camera, and a software algorithm determines the position and orientation of the tracking target relative to the camera. Because the front-facing camera on the HoloLens is at a fixed location relative to the display, coordinates determined using the camera are directly related to display coordinates. The HoloLens software development kit (SDK) allows developers to access the camera’s spatial position and perspective [30]. Developers can utilize the camera hardware and software tools with computer vision algorithms to determine the poses of 2D images or 3D objects relative to the headset point of view. The Vuforia Engine is officially supported [31], [32], though developers can leverage other computer vision tools, such as OpenCV [33].

**FIGURE 3.**
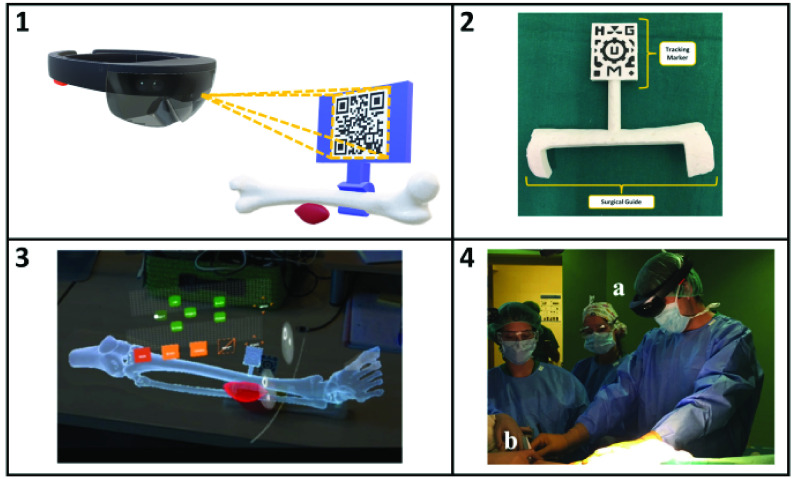
Schematic of a rigid registration guide with an image registration target (Panel 1). When the size of the image target is known, computer vision algorithms can determine the position and orientation of the image relative to the device’s front-facing camera. The guide is designed to attach to the patient anatomy at a single known position and orientation. This approach was applied to a 3D-printed surgical guide designed to attach to the tibial bone of a patient with Ewing’s sarcoma (Panel 2). Holographic anatomy was visualized by viewing the image target (Panel 3). The approach was evaluated during surgery (Panel 4) by a surgeon wearing a HoloLens headset (a) placing the guide (b) on the patient. Panels 2–4 adapted from Moreta-Martinez et al 2018 [34].

The computer vision capabilities of 3D-AR devices can be leveraged for registrations by placing computer vision targets at known locations relative to clinical workspaces or patient anatomy. This approach is very flexible and has been used in a wide variety of applications. One application of this approach utilized a patient-specific 3D-printed registration guide [34] (**Fig. 3**). The registration guide was a 2D image tracking target that was designed based on pre-operative imaging to rigidly attach to the patient’s tibia only in its precise, intended location. Because the guide’s position relative to the anatomy was determined by its physical design, the location of the pre-operative anatomy could be derived from the computer vision localization of the image target attached to the guide. Other clinical applications of image registration targets include a total hip arthroplasty case with a registration object attached to a bony landmark [35], occlusal splints attached to the mandibles of dogs used for 3D-AR-guided drilling [36], 3D-printed registration guides attached to the spinous processes of cadaver spines [37], and an orthopedic surgery simulator [38]. Another application of this technique utilized multiple computer vision targets at locations corresponding to fiducial markers worn during an MRI scan. This allowed the image data to be registered to the patient and viewed through the headset to aid in marking the location of breast tumors [39].

**FIGURE 4.**
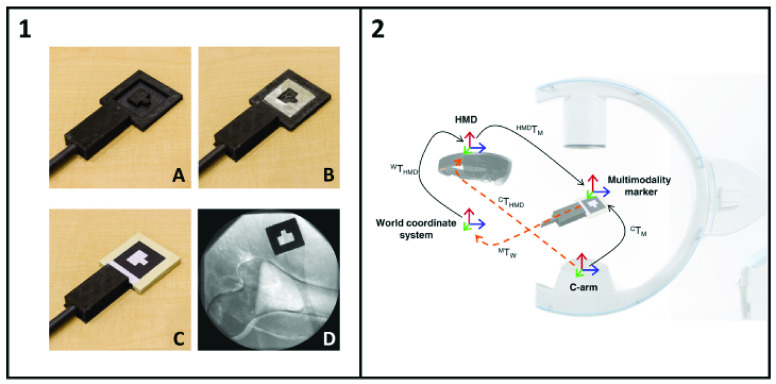
A unique AR registration approach utilized a multimodal fiducial (Panel 1). A marker pattern was 3D-printed (A) and filled with metal to create a radiopaque marker (B). A printout of the pattern that could be visualized by front-facing AR cameras was overlaid onto the marker (C). Because of the metal filling, the pattern was also visible in x-ray images (D). The marker was used to relate C-arm x-ray images to the headset coordinate system (Panel 2). Adapted from Andress *et al.* 2018 [41].

A computer vision target was attached to an endoscope as part of an Augmented Reality Assistance for Minimally Invasive Surgery (ARAMIS) system [40]. The marker and a 3D-AR headset allowed users to view a 3D representation of the workspace during a peg transfer task for laparoscopic skill evaluation. Another creative application of a computer vision registration target was a multimodal target for registering the headset with an x-ray fluoroscopy machine [41] (**Fig. 4**). The target was 3D-printed and filled with metal so that its pattern would be visible when viewed in x-ray images. The same pattern was then printed on paper and overlaid onto the metal target. This allowed the target to be visible to both the HoloLens and the fluoroscopy device. Registering the headset to the fluoroscopy coordinates allowed annotations on the 2D x-ray images to be displayed as 3D holograms that provided surgical guidance. Using this AR system, the investigators were able to substantially reduce the number of x-ray image acquisitions required to guide surgical instruments to targets while maintaining similar spatial accuracy.

## IV. Drift Compensation

Image registration targets are also useful for mitigating the effect of spatial drift in 3D-AR applications. Drift occurs because of limitations in the accuracy of 3D-AR headset spatial mapping systems. An early study on the suitability of 3D-AR hardware for clinical applications quantified spatial drift [42]. Study participants digitized four corners of a hologram before and after performing clinically relevant actions designed to challenge the spatial mapping stability. The actions included sudden head movements, walking, and temporary occlusion of the workspace. The authors found that these movements caused mean displacement errors of around 6 mm.

The effect of computer vision tracking on hologram stability was evaluated in a study using a 3D-printed skull phantom [43]. This study evaluated perceived hologram drift and hologram localization accuracy with and without an image target used to stabilize the hologram locations. The study used the Vuforia SDK and a feature that allowed the 2D image target to be wrapped around a cylinder to enable tracking from a wider range of angles. The addition of the image target decreased mean drift from 4.39 mm to 1.41 mm and improved point localization accuracy from 5.43 mm to 1.92 mm.

## V. Improving Localization Precision

**FIGURE 5.**
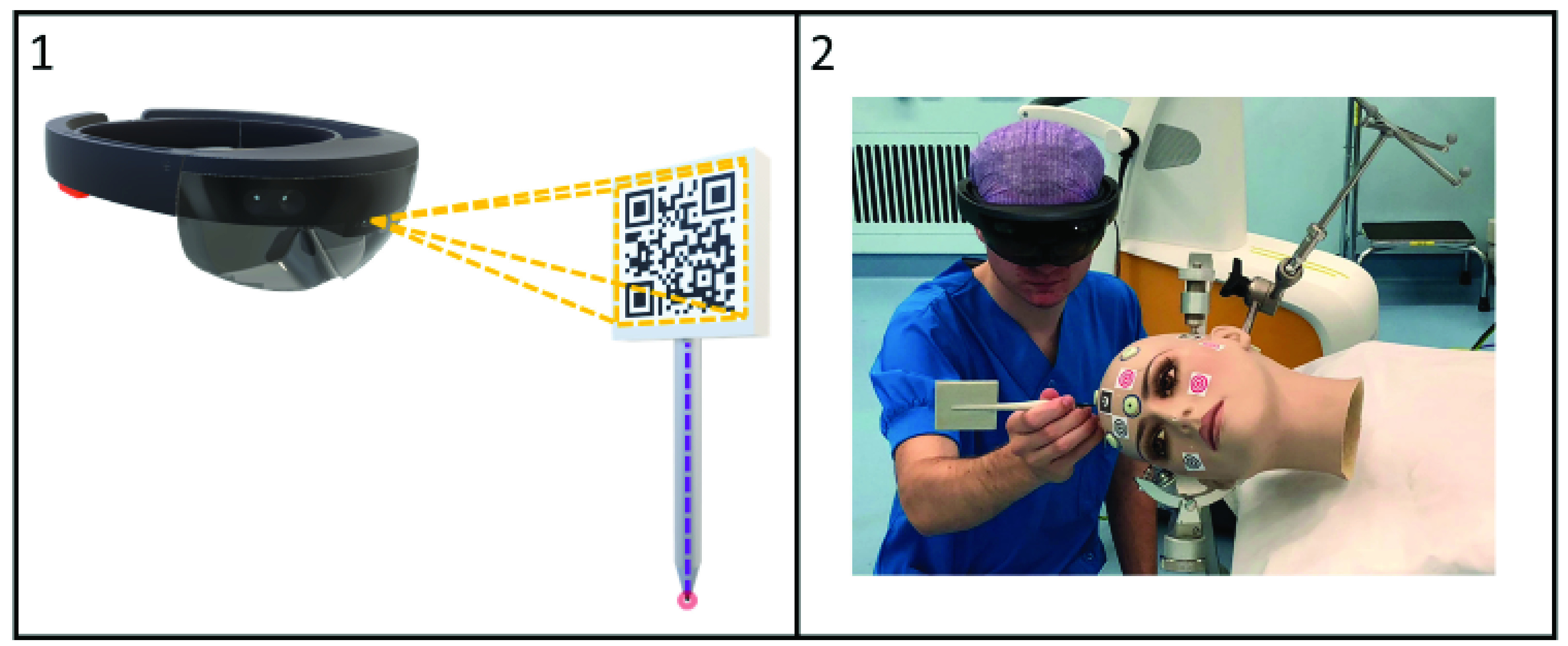
Precise localization of 3D points with AR hardware is difficult due to limitations in the spatial mapping accuracy. One approach to overcome these limitations is to attach a computer vision tracking target to a pointer of known geometry (Panel 1). The location of the pointer tip relative to the headset can be determined by tracking the image target. This technique was used to digitize fiducial points and register holographic anatomy to a plastic head model (Panel 2, adapted from van Doormaal *et al.* 2019 [44]).

In many cases, registration of hologram to patient anatomy requires precise 3D localization of anatomical landmarks or externally attached fiducial markers. Digitizing 3D points using the HoloLens can be achieved without any external hardware by taking advantage of the SLAM functionality. The SLAM functionality creates spatial maps of the environment, and the user can record 3D coordinates by pointing the visual gaze cursor at a location and performing a voice command or hand gesture to record the intersection of the visual gaze with the surface the user is looking at. However, the spatial maps of the environment generated by current-generation 3D-AR devices are optimized for flat surfaces such as walls, ceilings, and tables, and they cannot represent patients or clinical tools with the accuracy required for most clinical applications. Therefore, additional measures must be taken to stabilize holograms and register images to patients. One solution used to digitize 3D coordinates with the HoloLens headset was to affix an image tracking target to a digitizing pointer of known geometry [44] (**Fig. 5**). Because the location of the tip relative to the corners of the image target was known, the pointer could be used to record 3D coordinates with greater precision than would be possible using the built-in SLAM functionality.

## VI. External Tracking Hardware

While the built-in SLAM and computer vision capabilities of 3D-AR devices are useful for many clinical applications, external tracking hardware is often required to improve the precision of spatial tracking and localization. For example, one study evaluated 3D-AR for guiding a flexible needle to a 2 cm phantom target [45]. Precise tracking and registration were important because the application required tracking the needle base, and small errors in needle tracking or headset registration would cause much larger errors in the display of the needle tip.

Two common systems used for high precision tracking and localization are optical and electromagnetic (EM) tracking systems [46], [47]. Optical and EM tracking systems are both capable of tracking objects with sub-millimeter accuracy, though they have different trade-offs. In general, optical tracking systems can track objects in room-sized environments but require line-of-sight to tracked objects. In contrast, EM tracking systems can track objects without line-of-sight (including inside the body) but generally have smaller tracking volumes, and metal or ferromagnetic objects can interfere with EM tracking accuracy.

One way to incorporate external tracking systems into 3D-AR applications is to affix hardware to the headset to simultaneously track the headset and surgical tools or other objects that will be visualized in the headset. The primary challenge to this approach is that accurate hologram visualization depends on the position and orientation of the headset view origin, which cannot be precisely determined by physically examining the headset. When tracking hardware is attached to the headset, the coordinates of the tracking markers, **M**, are related to the coordinates of the headset view origin, **H**, by a rigid transformation with rotation **R** and translation **t**:

\begin{align*} {}^{\boldsymbol {M}}\boldsymbol {T}^{\boldsymbol {H}}=\left [{ {\begin{array}{cccccccccccccccccccc} \boldsymbol {R} & \quad \boldsymbol {t}\\ {0}^{T} &\quad 1\\ \end{array}} }\right]\tag{1}\end{align*}

**Fig. 6.**
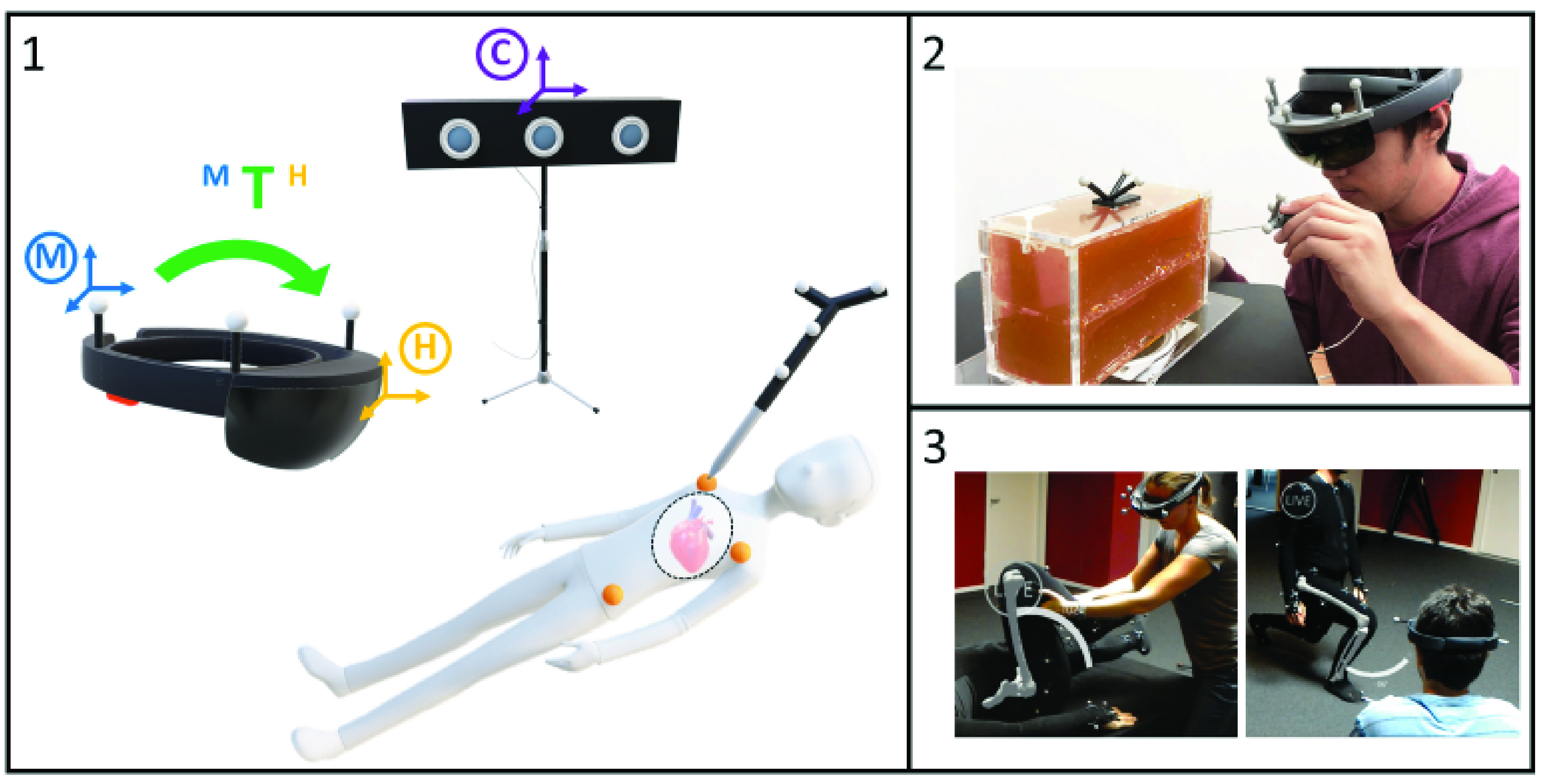
Incorporating external tracking into holographic software (Panel 1). An external infrared (IR) camera tracks objects with passive IR markers (white spheres) in a coordinate system local to the camera (C). The headset can be tracked using IR markers, however, the position and orientation tracked are relative to the markers affixed to the headset (M). The location and orientation of the headset coordinate system origin (H) is fixed relative to M, however, the view origin cannot be directly determined. A rigid transformation, T, mapping M to H must be solved for to incorporate camera data into the holographic software. Once the transformation is known, digitizing probes can be used to obtain fiducial or landmark positions and display holograms of pre-operative imaging data overlaid on the patient. Applications of this registration approach include real-time AR guidance for needle placement (Panel 2, adapted from Lin *et al.* 2018 [45]) and real-time skeletal visualization during human movement (Panel 3, adapted from Debarba *et al.* 2018 [49]).

This relationship is illustrated in **Fig. 6**.

Some studies utilized the HoloLens spatial tracking to solve for the transformation from headset trackers to view origin. An application of 3D-AR to remotely controlled spray painting used a four-second calibration where the HoloLens was moved in 3D space while tracked by an optical camera system [48]. The position and orientation data from the HoloLens and tracked data were matched during the acquisition and used to solve for the rigid transformation. A similar approach collected HoloLens and IR tracking motion data for calibration using a two-step process [49]. First, the HoloLens was translated in three axes while avoiding rotations. Next, the HoloLens was rotated about a spherical joint. This allowed the investigators to solve for the transformation from the HoloLens view origin to the optical tracker attached to the headset. While using the headset spatial tracking to register it to external tracking can be fast and simple, this approach does have limitations. The first limitation is that these registrations are affected by errors and drift in the spatial mapping system of the headset. Another limitation of these approaches is that they can require larger tracking volumes.

While optical tracking systems track large volumes well, registrations involving EM tracking systems tend to favor approaches that involve touching holographic points with a tracked pointer [50], [51]. In a study of a holographic view for catheter position during cardiac procedures, a catheter tracking system was registered to a HoloLens by touching virtual points with a magnetically tracked pointer [50]. To overcome the lack of tactile feedback and visual occlusion while physically touching holographic points, the registration was performed axis-by-axis for each landmark so that accurately judging hologram depth was not required.

An alternative approach to solve for the transformation in Equation 1 is to use a computer vision target with a known position and orientation in the external tracking coordinate system. This can be achieved by attaching optical or EM tracking hardware to the computer vision target, or by digitizing the location of the target using a tracked pointer. Using this approach, the registration between the 3D-AR headset and the external tracking system can be viewed as a hand-eye calibration. Originally developed for robotics applications [20], [21], this approach is described in detail and validated in a study of 3D-AR applied to orthopedic surgery [52]. This was also the approach used in the study of 3D-AR for guiding a flexible needle to a 2 cm phantom target [45].

## VII. Depth Cameras

In some cases, anatomical landmarks or registration fiducials are not available for registering pre-operative imaging to a patient. One solution to this problem that has been explored is the use of depth cameras. Depth cameras can be used for mapping and registrations by extracting sets of 3D points from imaged surfaces. Depth maps of head phantoms have been registered with surfaces generated from CT data [53], and image targets have been integrated into this setup to enable registration between the depth camera coordinates and a HoloLens headset [54]. Another study extended this idea by mounting the depth camera to a robotic arm [55]. The robotic arm allowed the camera to move around visual obstacles that could obscure its view of the target during surgery.

## VIII. Registration Accuracy

A key question concerning the use of 3D-AR guidance for clinical procedures is: what are the accuracy limits of these registrations? Unfortunately, because holograms lack tactile feedback and visual occlusion depth cues, quantifying the accuracy of these visualizations is not straightforward. A variety of different approaches for measuring registration accuracy have been explored. The “mixed reality capture” feature of the HoloLens has been used for quantifying registration accuracy [49]. This feature uses the front-facing camera of the HoloLens to record videos that capture the user’s physical surroundings and the holograms that were displayed by the headset [56]. The video frames can be analyzed to measure distances between holograms and their targets. While this approach is objective, multiple groups have noted that holograms can appear misaligned in mixed reality captures even when users perceived accurate alignment [43], [57]. These studies concluded that mixed reality captures are unreliable for quantifying error.

**FIGURE 7.**
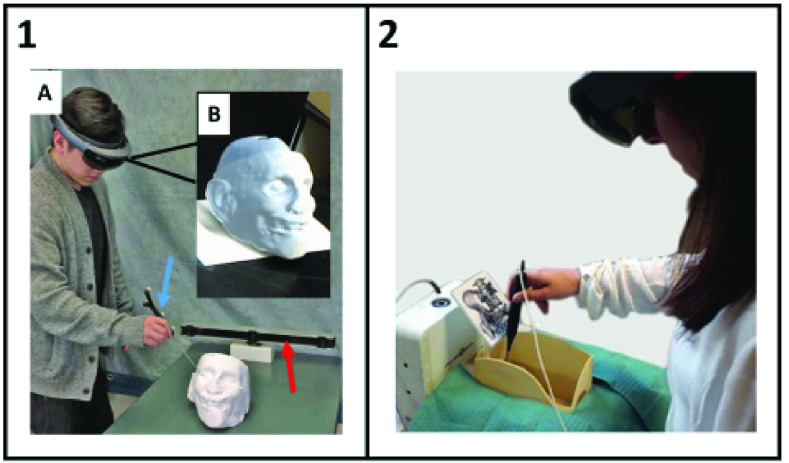
Assessments of hologram registration accuracy often evaluate users guiding tracked pointers to holographic targets. In one example (Panel 1, adapted from McJunkin *et al.* 2018 [25]), a user wearing a HoloLens (A) touched physical points on a 3D-printed head model (B) with an optically tracked pointer. The physical model was then removed, and the user touched the corresponding holographic points using AR guidance. In another approach, holographic targets displayed directly on a physical model (Panel 2, adapted from Condino *et al.* 2018 [38]). The accuracy measurement was based on how closely the user touched the intended locations with an electromagnetically tracked pointer.

Other registration accuracy assessments generally require human perception. These evaluations often measure the accuracy of the user when performing a clinically relevant task with 3D-AR guidance. While these assessments do not purely measure the quality of the AR visualization, what they do capture—the ability of users to perform clinically relevant tasks with AR guidance—is ultimately the most important metric. One common method for assessing registration accuracy is to use a tracked pointer to touch holographic targets and measure the distance error from the intended target (**Fig. 7**). Another method used to test the accuracy of 3D-AR guidance in multiple studies was to use the display for drilling guidance. The distance and angular deviation between the planned and actual drilling were used to assess the quality of the AR guidance. A summary of registration techniques, accuracy assessments, and accuracy measurements is provided in **Table 2**.

**TABLE 2:** Summary of 3D-AR Registration Study Approaches and Accuracy Results

Study	Clinical Focus	Hardware	Registration Method	Accuracy Evaluation	Accuracy
McJunkin et al. [25]	Lateral skull base anatomy, dissection, and image-guided surgery	HoloLens only	Hologram manually registered to physical target using voice and hand gestures	Physical object removed after registration and holographic targets touched by user with optically tracked pointer	Distance Error (Mean ± SEM): 5.76 ± 0.54 mm
Li et al. [29]	External ventricular drain (EVD) insertion for reducing intracranial hypertension	HoloLens only	Hologram manually registered to patient using electrocorticography electrodes as landmarks	AR-guided EVD insertion compared to conventional EVD insertion using distance error and passes required as accuracy metrics	Distance Error (Mean ± SD): 4.34 ± 1.63 mm (AR Guidance) 11.26 ± 4.83 mm (Control)
					Passes Required (Mean ± SD): 1.07 ± 0.258 (AR Guidance) 2.33 ± 0.98 (Control)
Azimi et al. [28]	General registration approach	External optical tracking system	Optically tracked handheld cube aligned with holographic cube targets	Optically tracked handheld cube aligned with additional holographic targets following registration process	Distance Error (Mean ± SD): 4.03 ± 0.87 mm
Liu et al. [55]	Computer-assisted hip resurfacing	Robotic depth camera	Computer vision tracking target pose determined by HoloLens and depth camera to determine correspondence between coordinate systems	Guide holes drilled on femur phantoms using AR guidance	Distance Error (Mean ± SD): 1.90 ± 0.84 mm
					Angular Error (Mean ± SD): 2.06 ± 0.89°
Moreta-Martinez et al. 2018. [34]	Surgical intervention for extraosseous Ewing’s sarcoma	Patient-specific surgical guide	Computer vision tracking target on surgical guide used to register HoloLens	Users touched randomly distributed spherical AR targets on surgical phantom surface with tracked pointer	Distance Error (Mean): 2.90 mm
Condino et al. [38]	Patient-specific simulator for orthopaedic surgery	Patient-specific surgical simulator	Computer vision tracking target attached to model for HoloLens registration	Study participants touched virtual targets displayed on surgical model	Distance Error (Mean ± SD): 2.0 ± 1.1 mm
Jiang et al. [36]	Craniofacial surgical navigation	Animal-specific occlusal splints	Computer vision marker attached to occlusal splints used for HoloLens registration	Surgical holes drilled in mandibles using AR guidance	Distance Error (Mean ± SD): 1.29 ± 0.70 mm (Entry) 2.47 ± 0.66 mm (End)
					Angular Error (Mean ± SD): 0.97 ± 2.89°
Müller et al. [37]	Vertebral pedicle screw insertion	Commercially available fiducial registration markers attached to spinous processes	Registration markers were equipped with titanium spheres and computer vision markers for registration with fluoroscopy and HoloLens, respectively	Cadaver spines instrumented by pedicle screws using AR guidance and conventional guidance	Distance Error (Mean ± SD): 3.4 ± 1.6 mm (AR Guidance) 3.2 ± 2.0 mm (Control)
					Angular Error (Mean ± SD): 4.3 ± 2.3° (AR Guidance) 3.5 ± 1.4° (Control)
Frantz et al. [43]	Neuronavigation	HoloLens tracking with computer vision marker to correct for drift	Hologram manually registered to phantom	Holographic markers touched with fine-tipped stylus	Distance Error (Mean): 1.92 mm (Drift Compensation) 5.43 mm (No Drift Compensation)
Condino et al. [62]	Accuracy limitations of AR guidance	HoloLens tracking with computer vision marker registration	Computer vision marker used for HoloLens registration	Participants performed 2D drawing tasks	Distance Error (Mean ± SD): 2.2 ± 0.8 mm (AR Guidance) 0.9 ± 0.2 mm (Paper Guidance)
Meulstee et al. [63]	Image-guided surgery	Optical tracking system with markers on HoloLens	Calibration performed to determine offset from optical markers on headset to HoloLens reference frame	Tracked object placed on 2D surface using AR guidance and compared to guidance using optical tracking system displayed on 2D screen	Distance Error (Mean ± SD): 2.3 ± 0.5 mm (AR Guidance) 0.7 ± 0.4 mm (2D Guidance)
Andress et al. [41]	Fluoroscopic x-ray guidance for orthopedic surgery	C-arm fluoroscopy machine	Computer vision marker filled with metal for x-ray visibility used to register HoloLens with fluoroscopy	K-wires placed using AR guidance vs fluoroscopic-only guidance	Distance Error (Mean): 5.20 mm (AR Guidance) 4.60 mm (Fluoroscopy Only)
					X-Ray Acquisitions (Mean): 5 (AR Guidance) 16 (Fluoroscopy Only)
Gibby et al. [70]	Image-guided spine procedures	HoloLens and optical tracker with IR and CT/MRI visible materials for dual registration	Manual and computer vision marker registration approaches used	Proceduralist guides needle to previously identified target site in preoperative imaging.	Distance Error (Mean ± SD): 1.73 ± 2.20 mm
Jiang et al. [71]	Vascular localization	HoloLens with computer vision markers	Computer vision markers used for HoloLens registration	Holographic points touched with tracked pointer (pointer supported by mechanical arm to eliminate hand tremor errors)	Distance Error (Mean ± SD): 2.85 ± 1.21 mm
Novarad Corporation 510(k) [17]	Device intended to visualize 3D imaging holograms of the patient, on the patient, for pre-operative localization and pre-operative planning of surgical options	Hololens SLAM + surface mesh registration.	Infrared time-of-flight depth sensor to generate surface geometry meshes of patient anatomy that can be registered with MRI or CT data collected previously	Hologram error measured with calipers using a regular pattern of spaced markers on a cube	Distance Error (Mean ± SD): 2.08 ± 1.86 mm
Augmedics Ltd. 510(k) [18]	Device intended as an aid for precisely locating anatomical structures in open or percutaneous spine procedures	Augmedics xVision with optical and radiopaque markers	Optical tracker single infrared camera using perspective n-point for registration	Observed screw orientation in post-operative CT imaging as compared to planned/virtual trajectory (as followed by proceduralist)	Distance Error (Mean ± SD): 1.98 ± 0.9 mm
					Angular Error (Mean ± SD): 1.3 ± 0.65°

While the accuracy assessment varies based on the clinical application targeted, there is consistency in the results. Manual registrations are generally the simplest to implement, though they also tend to be less accurate and introduce the possibility for human error. Based on the available literature, manual hologram registrations can achieve accuracies of ~4-6 mm. Because manual registrations rely on the built-in tracking capabilities of 3D-AR devices, their accuracies are ultimately limited by the 3D-AR device tracking. The addition of computer vision tracking targets increases the complexity of the registration but also increases the accuracy and stability. Studies that incorporated computer vision tracking targets often reported accuracies in the 2–3 mm range. The computer vision targets also serve to stabilize registrations, preventing errors due to accumulating drift in 3D-AR device tracking. Because computer vision algorithms are computationally complex, the performance of these approaches can be limited by the relatively low-powered computing hardware of most 3D-AR devices. Incorporating external tracking hardware offers additional advantages, particularly when tracking multiple objects or moving objects is required. Optical tracking systems can track in large volumes, and EM tracking systems can track without requiring line-of-sight. In a study comparing SLAM- and hand-eye calibration-based registrations with external tracking systems, de Oliveira and colleagues demonstrated that hand-eye calibrations that incorporate computer-vision targets drastically outperform SLAM-based registrations [52]. Studies using this approach also report errors in the 2-3 mm range.

## IX. Other Factors Affecting Accuracy

In addition to the method and quality of the registration, the user’s viewing angle and the quality of the AR visualization can affect AR-guided performance. The importance of viewing angle was demonstrated by a study that found that strictly perpendicular sightlines resulted in better accuracy than free sightlines when guiding a needle tip to a target with 3D-AR guidance [58]. Another important factor in 3D-AR visualization is the user’s inter-pupillary distance (IPD). In order to render 3D objects accurately on 3D-AR device screens, the user’s IPD must be taken into account. For a detailed treatment of how IPD measurement and errors affect 3D-AR visualization accuracy, we refer interested readers to existing work which has demonstrated that IPD errors can cause errors in depth estimation [59], [60]. The first-generation HoloLens IPD calibration consists of each display showing a number of targets and the user aligning a finger with the targets. The positions of the user’s finger and the transformation from headset sensors to the headset display are used to calculate the positions of the user’s eyes. In our own (unpublished) evaluation of the consistency of IPD calibration on the first-generation HoloLens, we found standard deviations of 1.57% of the mean IPD. The HoloLens 2 IPD calibration is a semiautomatic process that uses the headset eye-tracking functionality to detect the user’s eye position. Alternatively, the Magic Leap 1 relies on a manual process to directly measure the user’s IPD. It remains an open question whether improvements to IPD calibration accuracy will translate to more accurate AR guidance.

## X. Vergence-Accommodation Conflict

One possible source of error that may limit the accuracy of 3D-AR devices is the vergence-accommodation conflict (VAC). The VAC is caused by the optical distance of the displays in devices such as the HoloLens. When users look at virtual objects displayed in the HoloLens headset, their eyes must accommodate to a focal distance of 2 m [61]. Because the optical distance of the headset is fixed, this is true of all holograms, regardless of how far away they are intended to appear to the user. Because of this fixed focal distance for holograms, the user cannot visually focus on physical objects closer than 2 m and holograms at the same time. Consequently, when users perform motor tasks with AR guidance, they are forced to switch their gaze back and forth between different focal distances. One study attempted to quantify the error introduced by the VAC [62]. Users in the study used a ruler to draw lines connecting a series of dots. In one condition, the dots were drawn on a piece of paper, and in the other condition, the dots were displayed in an AR headset. The study found mean errors of 2.2 mm with AR guidance and 0.9 mm using paper. Maximum errors with AR guidance were 5.8 mm vs 2.7 mm with paper. These results are similar to another study in which users placed an object on a 2D working space using 3D-AR guidance in one condition and images on a 2D monitor in the other condition [63]. That study found mean errors of 2.3 mm with AR guidance vs 0.7 mm using the 2D monitor. Maximum errors were 3.6 mm with AR vs 2.0 mm with the 2D monitor. Whether the error was directly attributable to the VAC or was caused by some other aspect of the 3D-AR devices is difficult to prove, but it is interesting to note the consistency between these studies that found 3D-AR guidance added 1.3 mm and 1.6 mm of mean error, respectively, to manual tasks.

## XI. Psychophysical Issue

Another visual concern for some 3D-AR applications is the “psychophysical issue.” This issue arises when a properly registered hologram that is spatially behind or beneath a physical object appears to the user as being in front of the physical object [64], [65]. In the physical world, when one object is in front of another, the object in front occludes the object behind it, providing an unambiguous depth cue. Many applications of augmented reality are not compatible with the occluding behavior of physical objects. For example, holograms intended to guide surgical procedures may be overlaid on a patient’s skin but reveal the internals of an anatomical structure. The psychophysical issue can be mitigated by incorporating additional visual feedback about the position of the hologram relative to important physical objects. For applications with prior detailed knowledge of the occluding object, one study presented an efficient method to improve the depth perception of virtual objects relative to real objects by preprocessing an importance mask of the object [66]. Another study using pass-through video AR showed that using a non-photorealistic rendering of the overlaid object in addition to the overlay resulted in lower perceived depth errors compared to a standard overlay [67].

## XII. Improvements to 3D-AR Hardware

Clinical applications of AR will benefit from improvements to 3D-AR hardware. Compared to the first-generation HoloLens, the HoloLens 2 features a higher-resolution color video camera and higher-resolution time-of-flight depth camera. Additional improvements in hardware will likely emerge from the smartphone supply chain. Smartphones are the most widely available AR devices, and smartphone hardware has driven innovation in inertial measurement units (IMUs) and camera optics. The latest iPhones now feature light detection and ranging (LiDAR) hardware for depth-sensing. These tracking hardware improvements can directly impact 3D-AR applications that directly rely on 3D-AR device tracking, such as manual registrations. Improvements to the computational power of 3D-AR devices are also likely to move the field forward. Many aspects of registrations, including computer vision algorithms, are computationally intensive. The first-generation HoloLens had a 4-core 1 GHz CPU, while the HoloLens 2 has an 8-core 2.6 GHz CPU. The additional computational power combined with improved camera quality may enable an expanded role for computer vision tracking markers. This could include the use of additional markers, more stable tracking when the markers are farther from the device, or better performance when the markers are moving relative to the 3D-AR device. Display hardware is also rapidly advancing. The HoloLens 2 displays have a resolution of $2048\times1080$ pixels (per eye) compared to $1268\times720$ pixels in the first-generation HoloLens. The HoloLens 2 recently enabled auto eye position support [68]. This feature uses the on-board eye-tracking hardware to automatically determine the position of the user’s eyes relative to the displays. This may help reduce errors attributable to IPD inaccuracy and improve hologram positional accuracy, display quality, and user comfort. While we speculate that these hardware improvements will translate to improved results applying AR clinically, the quantitative impact remains unknown. New studies evaluating this hardware are needed.

## XIII. Conclusion

A growing body of literature supports the use of 3D-AR devices for guiding clinical interventions. The intuitive visualizations of 3D-AR devices have the potential to make difficult, skill-intensive procedures much more approachable. A study of 3D-AR guidance for CT-guided lesion targeted found that AR guidance elevated the performance of all users and helped novices perform as well as experienced clinicians [69].

A key challenge to using these devices in a clinical context is performing an accurate registration to the clinical workspace. One of the most accurate ways to register the 3D-AR devices to external coordinate systems is to use image targets and computer vision algorithms to determine the position of the target relative to the headset. This approach can correct for drift in the headset’s spatial mapping and can also be used to perform accurate registrations with external tracking systems. Evaluations of user accuracy when performing clinically relevant tasks suggest that accuracies of around 2 mm are feasible.

Error in 3D-AR device registration is due to contributions from sensor accuracy and resolution, manufacturing tolerances, human visual anatomy, and psychophysical effects. Improvements in hardware design will continue to make incremental improvements to the maximum attainable accuracy, while careful design can continue to mitigate human factors.

Competing Interests

The content is solely the responsibility of the authors and does not necessarily represent the official view of the NIH. Christopher M. Andrews, Alexander B. Henry, Ignacio M. Soriano, and Michael K. Southworth are employees of and hold equity in SentiAR. Jonathan R. Silva serves on the Board of Directors, a Consultant, and holds equity in SentiAR.
